# Biomarkers and Diagnostic and Therapeutic Approaches for Mycobacterial Diseases

**DOI:** 10.3390/tropicalmed11050139

**Published:** 2026-05-19

**Authors:** Arshad Rizvi, Yash Gupta

**Affiliations:** 1 Department of Chemistry and Biochemistry, University of Oklahoma, Norman, OK 73019, USA; 2 Department of Medicine, Penn State College of Medicine, Hershey, PA 17033, USA

Mycobacterial diseases, particularly tuberculosis (TB), continue to impose a substantial global health burden, disproportionately affecting populations in low- and middle-income countries [1,2]. Despite the availability of effective antimicrobial therapies, delayed diagnosis, prolonged treatment regimens, and the increasing emergence of drug-resistant strains remain major obstacles to disease control [3,4]. The growing incidence of extrapulmonary tuberculosis and non-tuberculous mycobacterial (NTM) disease adds substantial complexity to clinical management, as both often present with nonspecific manifestations and may mimic non-infectious conditions, thereby complicating timely diagnosis and appropriate treatment [5,6,7,8,9]. In our view, the persistent disconnect between early detection, definitive diagnosis, and therapeutic innovation remains one of the most understudied structural barriers in contemporary mycobacterial disease control.

This Special Issue, entitled “Biomarkers and Diagnostic and Therapeutic Approaches for Mycobacterial Diseases”, assembles five contributions spanning risk-based screening, diagnostic challenges in extrapulmonary and genitourinary tuberculosis, and therapeutic innovation through natural product discovery and machine learning-assisted drug repurposing. Together, these studies emphasize the need for integrated approaches that link early risk stratification with confirmatory diagnosis and diversified therapeutic development to improve outcomes across varied clinical and resource settings.

Addressing a central gap in latent tuberculosis infection (LTBI) risk stratification, Qiu et al. demonstrate the challenge of identifying individuals at risk of LTBI through routinely available clinical parameters. Using a retrospective cohort of college freshmen from tuberculosis high-burden regions in China, the authors explore associations between blood immune cell profiles, biochemical indicators, and LTBI status [10]. Their findings highlight eosinophil percentage and serum uric acid levels as factors significantly associated with LTBI, alongside well-established epidemiological risk markers such as prior tuberculosis exposure [6]. Importantly, the study proposes a prognostic model incorporating these indicators to estimate LTBI risk, underscoring the potential utility of accessible laboratory markers for targeted screening in high-risk populations [10]. Notably, such models will only possess clinical value if they are integrated into prospective screening frameworks rather than remaining as retrospective analytical tools.

Another contribution in this Special Issue highlights the diagnostic complexity of extrapulmonary tuberculosis through a case of peritoneal tuberculosis presenting with features highly suggestive of ovarian malignancy [11]. The authors describe a young woman with ascites, elevated tumor markers, and radiologic findings consistent with peritoneal carcinomatosis, in whom definitive diagnosis was ultimately established through histopathological and molecular analyses. This case underscores the capacity of peritoneal tuberculosis to closely mimic neoplastic disease, frequently leading to delayed diagnosis and invasive procedures [11]. By emphasizing the importance of maintaining a high index of suspicion, particularly in individuals from tuberculosis endemic regions, this report reinforces the need for multidisciplinary evaluation and careful interpretation of clinical, imaging and laboratory findings when assessing abdominal pathology in tropical and resource variable settings.

A further contribution within this Special Issue addresses the diagnostic challenge of genitourinary tuberculosis through a case of prostatic tuberculosis presenting with clinical, biochemical, and imaging features highly suggestive of prostate cancer [12]. In a tuberculosis endemic setting, the authors describe how elevated prostate-specific antigen levels and PI-RADS 5 lesions on multiparametric magnetic resonance imaging led to strong suspicion of malignancy, while histopathological examination ultimately confirmed tuberculous prostatitis [8]. By integrating a targeted literature review, this work highlights the substantial overlap between infectious and neoplastic prostate pathologies and underscores the limitations of imaging-based risk stratification tools in isolation [12]. The study reinforces the essential role of tissue diagnosis and clinical context in avoiding misdiagnosis and inappropriate management, particularly in high-burden, resource-constrained environments.

Beyond diagnostics, this Special Issue also addresses therapeutic innovation. One study advances antimycobacterial drug discovery through the identification of bioactive secondary metabolites from a newly characterized antibiotic producing *Streptomyces* sp. [13]. The study highlights the potential of natural product-based approaches to address persistent challenges posed by drug-resistant *Mycobacterium tuberculosis* and non-tuberculous mycobacteria. By demonstrating broad-spectrum antimycobacterial activity and low host toxicity, this work underscores the continued relevance of microbial secondary metabolites as a source of novel antimicrobial strategies [13]. Importantly, the identification of metabolite combinations with complementary biological activities, including those influencing host lipid metabolism, points toward innovative therapeutic paradigms that extend beyond single-agent antibiotics [13]. Collectively, this contribution reinforces the value of integrative metabolomic and functional screening approaches in expanding the pipeline for next-generation antimycobacterial therapies.

Complementing this approach, the final contribution employs a machine learning-assisted drug repurposing framework to identify compounds targeting essential mycobacterial enzymes, DNA gyrase A and serine/threonine protein kinase PknB [14]. Lee et al. integrate ligand- and structure-based screening with molecular dynamics to prioritize repurposed candidates targeting DNA gyrase A and PknB [14]. The identification of compounds with dual target potential highlights a strategic avenue for overcoming therapeutic limitations associated with single-target interventions and drug resistance [14]. More broadly, this work underscores the growing role of data-driven methodologies in modern antimycobacterial drug discovery and illustrates how computational innovation can complement experimental pipelines to streamline the development of next-generation tuberculosis therapies. However, computational prioritization must be matched by rigorous experimental validation if such approaches are to move beyond theoretical promise.

Looking forward, several research priorities emerge. First, biomarker-based LTBI risk models should be prospectively validated across diverse populations and epidemiological contexts, with attention to calibration, clinical utility, and implementation feasibility in resource variable settings [10]. Second, diagnostic algorithms for suspected extrapulmonary and genitourinary tuberculosis should be strengthened to reduce misclassification, integrating epidemiologic risk, standardized imaging interpretation, and timely histopathologic and molecular confirmation particularly when malignancy is a competing diagnosis [11,12]. Third, therapeutic development should prioritize rigorous mechanism of action studies, early evaluation of toxicity and pharmacokinetics, and standardized in vitro and in vivo validation frameworks to advance natural product leads and repurposed candidates toward clinical relevance [13,14]. Fourth, computational drug discovery approaches would benefit from reproducible benchmarking and clear experimental validation pathways (including target engagement and resistance accountability), ensuring that data-driven prioritization translates into clinically actionable candidates [14]. Addressing these priorities through coordinated clinical, laboratory, and computational efforts will be essential for improving the detection, classification, and treatment of mycobacterial diseases worldwide.

The field stands at a juncture where integration, rather than incremental advancement, will determine meaningful progress. Collectively, the contributions in this Special Issue reinforce the central premise that effective control of mycobacterial diseases depends on integrative frameworks that align clinical insight, diagnostic innovation, and therapeutic development.
